# Science, institutional archives and open access: an overview and a pilot survey on the Italian cancer research institutions

**DOI:** 10.1186/1756-9966-29-168

**Published:** 2010-12-20

**Authors:** Elisabetta Poltronieri, Ivana Truccolo, Corrado Di Benedetto, Mauro Castelli, Mauro Mazzocut, Gaetana Cognetti

**Affiliations:** 1 Publishing Unit, "Istituto Superiore di Sanità", Rome, Italy; 2 Scientific and Patient Library, "Centro di Riferimento Oncologico", IRCCS, Aviano, Italy; 3 Information Technology Unit, "Istituto Superiore di Sanità", Rome, Italy; 4 JECCR, "Istituto Regina Elena" National Cancer Institute, Rome, Italy; 5 Scientific and Patient Library, "Istituto Regina Elena" National Cancer Institute, Rome, Italy

## Abstract

**Background:**

The Open Archive Initiative (OAI) refers to a movement started around the '90s to guarantee free access to scientific information by removing the barriers to research results, especially those related to the ever increasing journal subscription prices. This new paradigm has reshaped the scholarly communication system and is closely connected to the build up of institutional repositories (IRs) conceived to the benefit of scientists and research bodies as a means to keep possession of their own literary production. The IRs are high-value tools which permit authors to gain visibility by enabling rapid access to scientific material (not only publications) thus increasing impact (citation rate) and permitting a multidimensional assessment of research findings.

**Methods:**

A survey was conducted in March 2010 to mainly explore the managing system in use for archiving the research finding adopted by the Italian *Scientific Institutes for Research, Hospitalization and Health Care *(IRCCS) of the oncology area within the Italian National Health Service (Servizio Sanitario Nazionale, SSN). They were asked to respond to a questionnaire intended to collect data about institutional archives, metadata formats and posting of full-text documents. The enquiry concerned also the perceived role of the institutional repository DSpace ISS, built up by the Istituto Superiore di Sanità (ISS) and based on a XML scheme for encoding metadata. Such a repository aims at acting as a unique reference point for the biomedical information produced by the Italian research institutions. An in-depth analysis has also been performed on the collection of information material addressed to patients produced by the institutions surveyed.

**Results:**

The survey respondents were 6 out of 9. The results reveal the use of different practices and standard among the institutions concerning: the type of documentation collected, the software adopted, the use and format of metadata and the conditions of accessibility to the IRs.

**Conclusions:**

The Italian **r**esearch institutions in the field of oncology are moving the first steps towards the philosophy of OA. The main effort should be the implementation of common procedures also in order to connect scientific publications to researchers curricula. In this framework, an important effort is represented by the project of ISS aimed to set a common interface able to allow migration of data from partner institutions to the OA compliant repository DSpace ISS.

## Background

### Introduction

"*Publishing exists to support research; research does not exist to support publishing"- Derek Law *[1]

Science publishing definitely represents a big deal. Market forecast in this field predicts millions of print and electronic journals as well as millions of customers, research staff, health personnel and public at large seeking for quality of health information. This generates a huge yearly turnover for commercial publishers. According to some studies carried out in the United States and cited by Danilo Di Diodoro [2], health expenses over the period 1986-1996 have raised by 84%, while the price of scientific journals increased by 148%, against an average increase of the recommended retail prices by 45%. This article is intended to reflect on crucial aspects of the publishing and archiving practice of research results by considering the authors' and research institutions' perspectives. Legal and economic issues concerning the production and dissemination of scientific content are faced together with the current solutions of publishing models based on the open access paradigm. The focus is centered on the habits and expectations of the search community acting in Italy in the oncologic subject area. In this regard, a survey offering an overview of the practices adopted by the Italian cancer research institutions to manage, organize and spread their research findings was conducted. The main goal of collecting data on these procedures (i.e. software used, metadata schemes, typology and contents of institutional repositories) is that of moving towards the adoption of shared technical standards (based on XML format) to encode data referring to scientific production (mainly publications). This will enable the aggregation and access to the scientific outputs produced by the Italian research institutions. The experience of the institutional repository DSpace ISS set up by the Istituto Superiore di Sanità is described as a promising tool to realize the objective of aggregating scientific content relating to the concerned domain. The merging of data referring to the scientific production of research institutions of the Italian National Health Service into the digital OA archive set up by the ISS, would guarantee the aggregation of resources and the wide retrievability of research results. In fact, institutional repositories as DSpace ISS, which adopt standard protocols to encode metadata, make online search engines able to capture their data thus enabling the harvesting process to disseminate contents on the net.

### Author's publishing practice and rights in a traditional journal system

What is a scientist supposed to do once his/her paper has been published in a journal? He/she, as the intellectual owner of his/her creative work, as well as the institution which has provided all the products and services required to support the scientist's work, are totally alienated from their own "creation". In contrast with all the laws regulating economy, the costs needed to product the goods are separated from profit. Not only the intellectual product is given away for free together with the all relating rights, but in many cases a journal may charge authors with publication fees. The assignment of copyright is required by 69% of publishers before the peer-review process, in which the publisher adds value to the scientific output. In this respect, it should be remembered that the referees too, in most cases, provide their advice for free. 15% of publishers even claim: "I reject your submission and do not grant permission to publish your work elsewhere". While 90% of publishers require the total assignment of rights, 6% claim for exclusive licenses and just 4% agree to subscribe for non-exclusive licenses [3].

This means that neither the author nor the institution are allowed to make papers freely accessible online, for example, by posting it on their own website or in a digital repository. They cannot even provide copies of the work to students during a course and not even the authors can share the work among colleagues. In addition to that, every single part of the article (i. e. tables or figures) cannot be reused by the authors without the permission from the publisher. The only way for both the author and institution to get access to the work is represented by the payment of a high-cost subscription to the journal in which the article appears. In this regard, if the subscription to *Brain research *is considered, it should be noticed that the amount to be paid in 1983 was 2,100 US dollars, while currently the charged subscription is over 20,000 US dollars. These costs are particularly burdensome for the less developed countries [3]. It often happens that libraries pay an institutional subscription in order to offer to its internal research staff free access to a collection of journals. But only the library is granted the permission, against the grain, from reluctant publishers to provide journal articles on exchange basis with other libraries. However, the condition imposed by publishers is that of delivering just the host made from flour to researchers - that is the printed copy of articles - to be taken once, and not the ordained host, the pure spirit, to mean the article circulating on electronic support to be easily taken and shared with other scientists, as in the holy communion.

### A paradigm shift: the implications of the open access publishing model

In the framework of the publishing process as a whole, is this organizing model still acceptable? In the Internet era the dissemination of scientific contents is mainly based on the use of online platforms superseding the strategy of commercial publishing used in the past to produce print journals and circulate them within the research community worldwide. At present, the innovative technologies of production and transmission of information in the net have generated models of scientific communication founded on the concept of free access to knowledge within a global context. In this regard, libraries, academies, learning societies and research institutions are increasingly committed to promote advocacy actions intended to gain free access to research findings - especially if resulted from publicly funded studies - beyond all types of barriers (technological, economic and legal ones).

This is the scenery in which the principles of open access publishing movement flourished. The scientific communication system starts to contrast the hegemony of commercial publishing and moves forward direct transmission of research results to the users (readers) by claiming free access to scientific knowledge, thus opening to a mechanism of disintermediation [4].

Briefly, open access literature is commonly recognized as synonym of free and unrestricted online availability of contents. A concise, but effective definition of open access is given by Peter Suber in "A very brief introduction to open access": *Open-access (OA) literature is digital, online, free of charge, and free of most copyright and licensing restrictions. What makes it possible is the internet and the consent of the author or copyright-holder *[5]. The OA movement started in 1991 thanks to the set up of ArXiv, the first repository of pre-prints in the field of physics. In 2001 the Open Archives Initiative Protocol for Metadata Harvesting (OAI-PMH) was created in order to define a standard procedure for unambiguously identifying metadata encoded in multiple formats, thus making repositories interoperable.

There exist two complementary strategies to achieve open access to scholarly journal literature: self-archiving which refers to the deposit of journal articles by the same scholars in digital archives compliant to OA standards (OA green route); publishing on open access journals which are freely accessible online but usually charge publication fees to authors wishing to publish on them (OA golden route). Both routes are stated in the *Budapest Open Access Initiative *(BOAI) launched in 2002 which represents a milestone of the open access movement. Other initiatives like the Bethesda Declaration and the Berlin Declaration in 2003 have occurred since the launch of the BOAI, all claiming free access to research output.

More recent perspectives of the OA movement were discussed during the seminar held in Granada in May 2010, *Open Access to science information: policies for the development of OA in Southern Europe *[6], attended by the delegates (researchers and information specialists) of six Mediterranean countries of South Europe (France, Italy, Turkey, Greece, Portugal). This seminar stressed the importance of the following actions: link the open digital archives to the *National Research Anagrafe*; guarantee high quality standards of the OA journals; reduce the cost of publications by moving from the paper to the digital publishing; define common standard to facilitate the gathering and aggregation of metadata.

Moreover, a new service announced at the *Berlin 8 Conference on Open Access *held in Beijing in October 2010 and intended to implement OA strategies is about to be launched by OASIS (Open Access Scholarly Information Sourcebook) in 2011: *The open access map *[7] a world map and chronology which shows all OA projects, services, initiatives and their development over the last ten years.

### Open access in Italy

As far as Italy is concerned, an important breakthrough for the academic world was marked by the Messina Declaration, in 2004, the first institutional action on the part of the chancellors of the Italian universities in favour of OA. This event represented the starting point of an action towards the statement of policies requiring researchers to deposit their papers in institutional repositories and to publish research articles in OA journals.

Among the most recent Italian initiatives aimed at promoting the OA philosophy, it is worth mentioning the launch in 2008 of the *Italian wiki on open access *[8], conceived as a reference point on Italian projects and best practices. Another reference point is also the DRIVER wiki containing a section devoted to *Open access in Italy *[9] while the state of the art of the OA initiatives is described in *Open Access in Italy: report 2009 *offering a wide overview on the ongoing projects and experiences [10].

### Open access in science and medicine

A decisive impulse to the unrestricted availability of research results (scientific publications and data sets) is represented by the OpenAIRE Project (Open Access Infrastructure for Research in Europe) [11]. This Pilot Project, financed by the European Commission and covering the 27 member states of the European Union, has been conceived to deliver both a technical and a networking infrastructure to the benefit of the research community. The former infrastructure is aimed at collecting and providing access to the research articles reporting on outcomes of FP7 and European Research Council (ERC) projects, while the second one, based on the creation of a European Helpdesk System, has been designed to best support the practice of archiving in each EU member state.

Another ongoing project centered on the strategy of linking experiences and innovations under the umbrella of OA access to quality health information is NECOBELAC (Network of Collaboration Between Europe & Latin American-Caribbean countries) [12,13]. Its core objective is to raise awareness on the benefits of open access to public health information. The Project was funded in 2009 by the European Commission under the seventh Framework Program and is led by the Istituto Superiore di Sanità. The Project aims at creating a network of institutions in Europe and LAC countries which collaborate to provide training programs on the themes of scientific writing and innovative publishing models, based on immediate, open, and permanent access to research findings.

Along with the spread of OA initiatives, some commercial publishers gradually realized that the traditional publishing system would have no chance of survival thus leading, sooner or later, to a financial crisis in scholarly publishing industry. Therefore some open-access publishing pioneers as BioMed Central (BMC) decided to adopt new market strategies as that of replacing subscription charges to scholarly journals with article publication charges. This implies that the author is recognized as the copyright owner in the published text, and the scientific works become quickly available online for all to read, download, print and distribute, provided that the work's integrity and the author's intellectual property is respected. BMC, along with many other OA publishers, has joined the Open Access Scholarly Publishers Association (OASPA) [14] which has adopted a Code of conduct to whom all members are expected to adhere. This means that authors wishing to publish on OA journals issued by the publishers associated to OASPA can benefit from a tool which ensure quality standards in the OA publishing sector.

Some traditional publishers as Oxford University Press, which publishes *Annals of Oncology*, offer an hybrid model which, besides the usual subscription one, foresees the option to pay a supplementary fee in order for the author to maintain the ownership of the copyright in the published work.

Many publishers have therefore been forced to give up under the pressure of the OA movement, thus allowing free self archiving of pre prints (author's manuscript version before peer review) together with post prints (final author's version after peer review, but not always the publisher's Pdf) even though in some cases a period of embargo from the publication date of an article is envisaged. Authors can check publishers' policies concerning conditions and restrictions for the self archiving of their papers by browsing the service RoMEO (*Publisher copyright policies & self-archiving) *[15] or *Journal Info *[16]. Currently, over 90% of publishers let authors manage their own papers by allowing free deposit of works in institutional repositories.

### Institutional repositories as pioneers in the open access arena

On the role of institutional repositories (IR) in pursuing the free and timely distribution of scientific information, it is worth mentioning the activity of the Conference of Chancellors of Italian Universities (Conferenza dei Rettori delle Università Italiane, CRUI), through its Open Access Group acting within the Library Commission, which has recently established *Guidelines on the establishing of academic institutional repositories *[17].

The issue concerning the institutional repositories is intimately related to the concept of free access to research results to increase visibility, impact and sharing of scientific information. Academic and research institutions worldwide increasingly adhere to the open access paradigm through the establishment of institutional repositories aimed to fully maximize the visibility of their research outputs. The two main tools collecting timely data on the number of such digital archives are the *Registry of Open Access Repositories (ROAR) *[18] and Open*DOAR*, *Directory of Open Access Repositories *[19] respectively count 2049 and 1815 installations all over the world. Visibility and impact of repositories are also constantly monitored by using web indicators as shown twice a year (January and June editions) on the *Ranking Web of World's Repositories *[20]. The building-up and maintaining of the institutional repositories foster close interaction between diverse categories of professionals: the information specialists dealing with the quality control and standardization of bibliographic data, the data management experts designing the workflow of data handled by the users, the institutions' managers (administrators) defining official policies and the researchers providing their papers to be posted to the repositories (self-archiving procedure). Digital repositories complying with the standards set by the Open Archives Initiative (OAI) [21], are called "interoperable"; interoperability is the capability of exchanging data aiming to facilitate the efficient dissemination of content. This means that users can find their contents without knowing which archives exist, where they are located, or what they contain. OAI-compliant archives are based, built and maintained on open-source software. Such digital containers give great visibility to scholarly literature on the web; this is proved by the fact that the traditional search engines, as Google, present them as first results of the queries launched by the users.

Institutional repositories, as digital containers of research output, have definitely to be conceived as strategic tools to manage, spread and preserve research information within an institution. They essentially work as stable windows online to timely show up the resources produced by the scientific community. In this respect, the awareness of researchers as authors and readers of scientific literature is fundamental, as each individual publication is by now, in the Internet era, part of a global information network. Repositories, in fact, nowadays often represent only means of performance appraisal used for the distribution of research funds. This perceived bias may generate suspicious about the real objective of such tools, that is to enhance the global access to scientific information.

The institutional repositories built up to storage the scientific literary production of the research bodies in Italy are mainly intended for evaluation purposes in view of the annual activity report and for assigning funding to research investigations. They are not properly used, as they should be, for their characteristics of information richness meant to provide high visibility to the national scientific output and to enable to search for scientists competences and specializations. There should be a need for promoting these digital archives through governmental policies as they definitely represent fundamental tools for integrating free access scientific resources at national level. As far as the production of research literature in Italy, it should be considered that it is retrievable thanks to powerful indexing services as PubMed managed in the US. So there is great expectation regarding the development of digital archive dedicated to the Italian research in the field of public health. Such a realization may represent the solution to overcome the gap between Italy and other countries which can rely on already existing centralized services. ISS DSpace could permanently store and make accessible worldwide online the national scientific production.

## Methods

### Open information tools in the health sector in Italy

As far as the existence of OA compliant repositories set up by biomedical research institutions in Italy, the scenario is still poor. A research performed on OpenDOAR, in December 2010, resulted in just four repositories managed by Italian institutions classified under "Health and Medicine", over 59 Italian repositories indexed by the Directory: *E-ms (Archivio Aperto di Documenti per la Medicina Sociale), Ilithia (Università Campus Bio-Medico di Roma), Istituto Superiore di Sanità Digital Repository (DSpace ISS) and Open Archive Siena (OASi). *No matches were found in the same period by launching a query in ROAR Advanced search by combining "Medicine" as subject and "Italy" as country, over 62 Italian repositories indexed by the Registry. DSpace ISS is indexed as Research Cross-Institutional under the class "Repository type" in ROAR. Anyway, leaving apart the results of the search by subject area that could be biased by the fact that the repositories set up by universities are multidisciplinary, the majority of them, sorted by "Italy", belong to universities and not to research institutions.

The figures concerning the OA journals searched in DOAJ in the same period (December 2010) resulted in 63 journals ranked under "Oncology" of which just two titles resulted as issued by Italian publishers: *Haematologica *and *Rare Tumors.*

The research community of oncologists in Italy take advantage of a recognized source represented by the official journal of the "Regina Elena" National Cancer Institute in Rome: the *Journal of Experimental & Clinical Cancer Research (JECCR)*, founded in 1982. In 2008, in order to offer a more rational and cost-effective system for scientific communication, the JECCR became an open access online publication, published by BioMed Central (BMC). It, as already said, is an independent publishing house committed to providing immediate open access to peer-reviewed biomedical research and was chosen on the basis of its prestige as witnessed by over 180 online open access journals covering the whole of biology and medicine.

Moving from traditional printed copy to online editing, represented for the Journal a quantum leap in terms of: number of annual submissions (over 70%); rapid publication and higher visibility (from nine to three months from submission to PubMed, with consequent increase of the citation ranking); in particular the immediacy index (impact factor computed in the same year of publication) has grown from 0,048 in 2007, to 0,127 in 2008, reaching 0,308 in 2009.

Also the manuscript tracking during and after the publication process, for instance the number of times the article is viewed or downloaded is more and more growing. In conclusion, the *Journal of Experimental & Clinical Cancer Research *experience confirmed that online open access ensures a wider dissemination of the research accompanied by a good cost-effectiveness.

As far as the information tools addressed to lay people, an interesting open access resource in the field of oncology and public health is represented by Cignoweb.it [22]. It consists in an online data bank conceived for the benefit of patients, their families and the general public, and is based on a Project coordinated by the Centro di Riferimento Oncologico (CRO) of Aviano, in collaboration with the ISS, the Istituto Farmacologico Mario Negri of Milan and Medinfo (Laboratorio di nanobiotecnologie e informatica medica) for software implementation. Cignoweb.it is part of a wider project supported by Alliance Against Cancer [23] aimed to set up in Italy the *National Service for the Welcoming and information *with the collaboration of the *Italian Cancer Voluntary Association Federation *(FAVO). In particular, Cignoweb.it intends to achieve the following objectives:

1 - Check for all information material in any support, produced in Italy and addressed to patients; assess the quality of the information retrieved and make it accessible on the web through a single, user-friendly and integrated interface;

2 - Make available an authoritative source of information to the benefit of the lay people, aimed at improving the communication between citizens and health facilities in Italy, thanks to the creation of reference points for the spread of information;

3 - Lower barriers to the access to reliable information for citizens-patients and contribute to promoting a culture based on the concept of a critical evaluation of information;

4 - Promote an appropriate use of the available services and resources in order to better tackle disease problems and make informed decisions face clinical trials or innovative therapies.

The software prototype has been just implemented and, at the moment, it allows for free access to resources and documentation based on paper, electronic or multimedia support. This information material is mostly in Italian and written in plain language and includes: booklets, brochures, articles, mailing lists, books containing testimonies relating to health facilities, associations and help lines, forums, blogs and social networks. The most of it concerns the subject area of oncology, but other fields of biomedicine are foreseen for inclusion. The distinctive feature of all material considered for indexing in Cignoweb.it is represented by the quality assessment performed on the entered material.

The Cignoweb.it editors hope that the prototype could support other European countries in enhancing the structure and organization of the patient health information produced in their own national languages. In this way, Cignoweb.it will contribute to support ideas and actions aimed at building a common health information portal in the European Union. In particular, Cignoweb.it is trying to collaborate with the EU project EUROCANCERCOMS [24]. This EU coordination and support action aims to establish an integrated model for a Europe-wide cancer information and policy exchange portal that will provide a functional exchange system for accurate information and intelligence, catering to the needs of health professionals, patients and policy makers. To address this, a consortium will conduct an inventory of all existing information tools, their faults and flaws and requirements for the future. Cignoweb.it represents the Italian contribution to the building of a European Area for Cancer Information.

### Standardized metadata for aggregating Italian biomedical publications

Repositories contain metadata, say "meta information" (data about data). They can be defined as structured data which describe the characteristics of a data set and how the data themselves are formatted. Metadata refer, for instance, to authors, abstract, subject, rights and other elements describing an item in a standardized format. According to Ed Simons "Metadata allow us to describe and classify research information in a systematic way, and as such they are indispensable for searching and finding academic publications and other results of research." [25]

In addition to traditional metadata (formal and content ones) commonly used in repositories, new types of metadata should be considered for inclusion: the context metadata. They add high value to the single lists of publications shown in a repository as they lead to discover all the information around a publication, for instance the institutions and the researchers involved, the research project, the publication results from, the funding program, patents etc. These additional metadata allow the user to surf the Internet from a link to another, starting from a single publication posted in a repository, to a researcher curriculum or to the data concerning the institution which produced the research or to other related data, thus enabling an effective navigation through different types of information. In order to fulfil this aim an important effort to be made is the standardization of different formats in use to describe the same item. So, it is relevant the adoption of thesauri for indexing the information by concept, but also the use of permanent identificators relating to authors or institutions. Beside the DOI (Digital Object Identifier) mostly used for articles, the DAI (Digital Author Identifier) and the DII (Digital Institution Identifier), already adopted by some European projects (CRIS/CERIF) may become relevant tools to mark data in a standardized way.

Context metadata are the core elements of the so-called citation based networks, the privileged domain of interest and activity of the communities working in a CRIS (Current Research Information System) environment. One particular type of CRIS standard for information systems is the CERIF (Common European Research Information Format) standard, proposed by the European Union and developed and maintained by euroCRIS. This relevant perspective for the future of repository technology was recently debated at international level during a Workshop organized by the *Institute for Research on Population and Social Policies *of the *National Research Council *(CNR), in Rome [26].

Turning to the ongoing Italian initiatives with metadata storage and supply in the biomedical field, the experience gained by the Istituto Superiore di Sanità is worth to be mentioned. In 2004 the ISS launched a project aimed at creating a digital archive compliant with the aims of the Open Archives Initiative. In 2006 the ISS built up its own repository, DSpace ISS based on the DSpace platform [27]. The primary object was to provide both data and services regarding research material produced by the ISS research staff. DSpace is an OAI compliant open-source software released by MIT (Massachusetts Institute of Technology, US) for archiving e-prints and other kinds of academic content. It preserves and enables easy and open access to all types of digital content including text, images and data sets.

The primary goals to be achieved were to store digital information and index it by assigning descriptive metadata in order to keep research material accessible and to preserve content in a safe archive, according to an internal policy (Institutional Policy for Open Access to Scientific Publications) available from the home page of DSpace ISS website. Content retrieval based on the adoption of MeSH terms in the indexing of DSpace ISS items has also featured the repository from the very beginning [28]. MeSH (Medical Subject Headings) is the thesaurus developed by the US National Library of Medicine, used by PubMed. MeSH descriptors are part of the Unified Medical Language System (UMLS), a relevant tool of controlled medical terminology enabling semantic search across more than a hundred standard sets of biomedical terms, and ensuring interoperability among different systems. MeSH have been translated into many languages and have become an international standard for indexing biomedical literature. The Italian MeSH translation, carried on by the Istituto Superiore di Sanità, is freely accessible online on the ISS website [29]. Moreover, the Italian MeSH translation has been adopted by many Italian research institutions for indexing and information retrieval purposes.

Basically the idea was to create a privileged reference point for online free access biomedical information produced by Italian research bodies. Therefore, in parallel to the installation of the repository, the ISS started developing partnerships with other research institutions operating within the *Italian National Health Service*. The aim was that of allowing partners supply their data and browse their own entries stored on the central DSpace ISS server. In this perspective, together with its own publications, the repository began to hold a selection of bibliographic data provided from partner institutions, most of which belong to Bibliosan [30], the *Italian Research Libraries Network*, a collaborative initiative conceived to spread health information and services and promoted by the Italian Ministry of Health. Thus, new communities and collections were gradually being created in the repository.

Due to the different metadata formats in use by the partner institutions, the ISS has recently implemented an XML schema, based on the Dublin Core metadata set. The main idea arose from the need to establish a workflow for migrating metadata from partner data files to DSpace ISS. A standard data format along with the completeness and consistency of data to be gathered from the DSpace ISS partner institutions will result in a more effective archiving of documentation in the ISS open repository [31]. This allows users to better retrieve the information and to enhance innovative methods for both monitoring and appraising of the scientific output produced by the Italian research community. Moreover, the adoption of common standard of metadata stored in different platforms would enable the interoperability with other open systems and with the CRIS/CERIF initiatives, as well as the automatic overflow of data in OA International archives as PubMed Central (the open archive of life sciences journal literature managed by the National Library of Medicine of Bethesda, US) thus optimizing the visibility of research findings to the scientific community worldwide.

The ISS is also working to set import and export options in DSpace ISS interface for data encoded in different formats. The current available option is the metadata uploading process through the XML schema defined by ISS for files encoded with the RefWorks software. *RefWorks*, as *Endnote *or *Reference Manager*, are bibliographic management programs used to format a large number of references, according to the different styles required from scholarly journals. This kind of software also provides direct export methods operating on the web to capture citations from external databases including the full text, when available. Due to their features and user-friendliness both for scientists and research managers, these systems could be very useful to manage bibliographic data stored in institutional repositories. Moreover, two of these programs, namely *RefWorks *ed *Endnote*, have been recently made available by the Network Bibliosan as new acquired services to the benefit of the whole staff of the research institutions of the *Italian National Health Service*. They provide possibility to import rich and various metadata from online databases as PubMed with no need for the repositories' manager to re-enter data. Quality and quantity of metadata represent fundamental features for the architecture of the open archives, being the key factors of system capacity to organize, manage and retrieve relevant information. As far as the available software that automatically generate bibliography, it would be useful to test open source product as Mendeley, a free reference manager with interesting features. The ISS has already implemented a software and is running a trial of its application with the Istituto Zooprofilattico delle Venezie and the Istituto Regina Elena of Rome in order to organize the migration of data encoded with *RefWorks *toward DSpace ISS. In addition to that, the ISS is collaborating with the Centro di Riferimento Oncologico of Aviano to test the uploading in DSpace ISS of data formatted with *Reference Manager*. Unfortunately, citation management software is still scarcely used to manage institutional repositories. This is the reason why, according to the needs of the Bibliosan community, the ISS has released a minimum data set of bibliographic metadata to allow the automatic download in DSpace ISS of the citations referred to the annual literary production of the institutions belonging to the Bibliosan network. This standard set of metadata is derived, with adaptations, from the format adopted by the Bibliosan institutions specifically intended to yearly report the scientific published works to the Italian Ministry of Health. This format is only conceived for providing administrative data useful for political decision relating to funding, so it is poor as far as bibliographic metadata are concerned.

**Figure 1:**
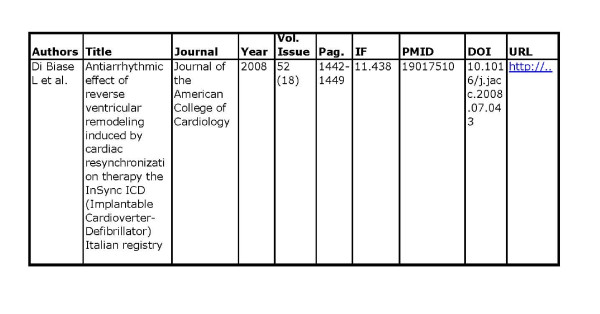
**Basic data set to be filled by partners institutions of DSpace ISS**.

**Figure 2:**
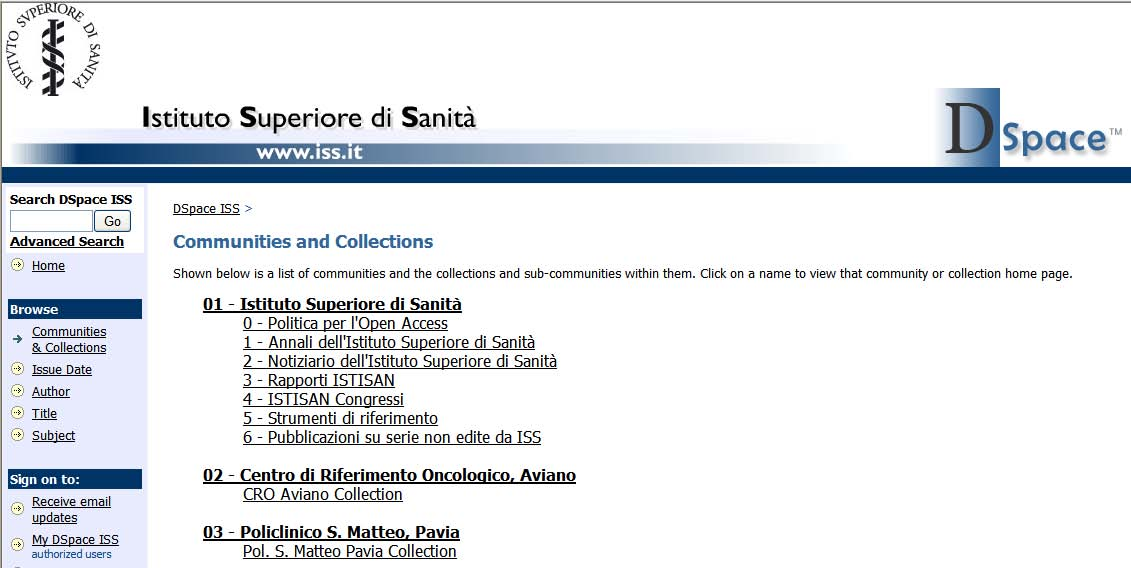
**List of some communities created in DSpace ISS**.

The minimum data set has been agreed by Bibliosan, (Figure 1). Data files (i. e. Excel files) from Bibliosan partners will be therefore downloaded in the ISS server to be then uploaded to DSpace ISS automatically (Figure 2). The minimum data set formulated for Bibliosan foresees the following metadata: authors (column A), title of the article (column B), title of the publication (column C) year of publication (column D), number of volume and issue (column E), pages (column F), impact factor value (column G): the metadata from columns A to F are mandatory in order to create the citation, whereas PMID (PubMed Identifier, column H), Digital Object Identifier (DOI, column I) and Unified Resource Locator (URL, column J) have been considered optional.

Referring to future initiatives, creating a workflow of data between DSpace ISS and the system run by the *Italian Ministry of Health *would mean to move forward the realization of a permanent free access point to the national scientific output, thus providing tools for a multidimensional evaluation of the resources produced. In this way, Italy could find its place within the context of the European countries which are investigating advanced management systems of research results.

### A survey of oncological IRCSS publications managing system

In March 2010 a questionnaire was administered to nine Italian cancer research institutes "Istituti di Ricovero e Cura a Carattere Scientifico" (IRCCS) acting in the field of oncology. These institutions are devoted to biomedical research to the benefit of the patients and to the medical community. They are: Istituto Tumori Giovanni Paolo II, Bari; Istituto Europeo di Oncologia, Milan; Fondazione Istituto Nazionale per lo Studio e la Cura dei Tumori, Milan; Istituto Nazionale per la Ricerca sul Cancro, Genoa; Istituto Regina Elena, Rome; Centro di Riferimento Oncologico, Aviano; Centro di Riferimento Oncologico della Basilicata, Rionero in Vulture; Istituto Nazionale Tumori Fondazione Giovanni Pascale, Neaples; Istituto Oncologico Veneto, Padua.

The questionnaire was e-mailed to the librarians of each institution. The survey was basically intended to identify: the archive holdings (type of research outputs contained in institutional repositories) and the system in use to support archive operations (software or paper-based system). Such information would serve the purpose of providing a baseline to explore the feasibility of a standardized workflow of data from partners joining DSpace ISS.

In the subject area of oncology, the Italian research institutions surveyed in this study represent a privileged point to go in depth with the analysis of strategies to collect and disseminate relevant information to the benefit of both the scientists and the general public.

## Results

### Responding institutions

The respondent institutions were six out of nine and precisely: Istituto Europeo di Oncologia, Milano; Istituto Regina Elena, Roma; Centro di Riferimento Oncologico, Aviano; Centro di Riferimento Oncologico della Basilicata, Rionero in Vulture; Istituto Nazionale Tumori Fondazione Giovanni Pascale, Neaples; Istituto Oncologico Veneto, Padua. As far as the Unit responsible for managing the publications, in three cases it was the " Scientific Direction", while in two cases it was the Library and in one both Units together.

### Type of archived material

**Figure 3:**
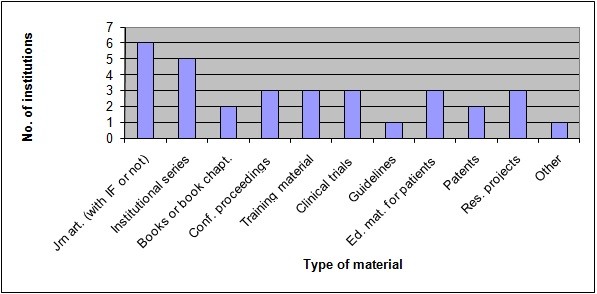
**Type of material included in the databases of the surveyed institutions**.

With regard to the type of material considered, all participants in the survey declared they archive journal articles, with or without impact factor (IF); five institutions out of six declared they describe their own series (consisting of journals, technical reports and newsletters). Conference proceedings were included in the material archived by only three institutions, as well as training material, clinical trials, information material addressed to patients and rationales or synthesis relating to research projects. As last, two respondents consider books or book chapters for inclusion in their archives, whereas just one institution includes guidelines and another one selected *Other *as a different type of material different from the mentioned ones in the questionnaire [Figure 3].

In the majority of cases (4 out of 6) the entries are represented by bibliographical citations; in 2 of them the full text is posted together with the bibliographical reference.

### Software used

All respondents answered they use an electronic system to manage the publications: both Word and Excel resulted the software adopted by three institutions out of six, whereas just one uses RefWorks, another one uses Reference Manager and the remaining one mentioned an in-house software ad hoc, not specified, and a not specified software tool.

### Metadata applied

**Figure 4:**
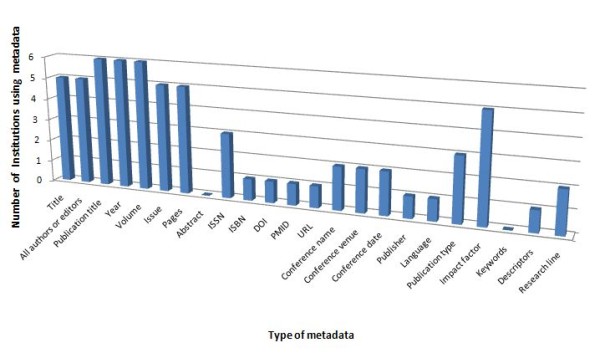
**Metadata used by the surveyed institutions**.

Respondents were also asked to indicate the metadata used to describe publications in their databases. In terms of quantity of metadata envisaged, the answers were variable. Only one institution selected almost the total of metadata listed on the questionnaire, including conference data: title, venue and date (Figure 4).

### Format of metadata

As far as the author's name, four institutions answered they enter both last and first names, one close to the other, in the author(s) field within a record, thus without envisaging separate fields for surname and first name. No answers on this point came from two institutions. The format for entering personal author name follows different rules: Rossi M; Rossi,M; Rossi, M.; Rossi M. (2 institutions). The problem of the standardization of the metadata format is relevant in order to permit a sound organization and a good retrieval of information, especially in the context of digital archives sharing metadata.

### Accessibility

Another indicator the participants in the survey were asked about was the level of accessibility to their publications databases. In this regard, four respondents said that only the "Scientific Direction" is allowed to access data, while in two cases the contents are available to internal researchers on Intranet.

### Institutional series

As far as institutional series published by the research centers participating in the survey, all of them, except one, experienced the production of reports, newsletters and other official information material made freely available online with the only exception of one institution which did not provide access to its ceased internal bulletin.

### Future developments

All institutions agreed to the proposal of sharing their publications in DSpace ISS by establishing communities and collections of their own documents; some of them (IRE and CRO) already joined the ISS digital archive.

The situation is in progress. While this article is going to press, the Istituto Regina Elena decided to adopt RefWorks for its own institutional archive, in order to set up a good collection of standard metadata and achieve a better organization of the archive.

## Discussion

Thanks to the existing online platforms, institutional policies mandating self-archiving in institutional repositories are definitely needed, mainly for papers describing research activity financed by public funds. This represents an ineluctable process as underlined by Stevan Harnad [32], one of the gurus of the open access movement: "The freeing of their present and future refereed research from all access- and impact-barriers forever is now entirely in the hands of researchers. Posterity is looking over our shoulders, and will not judge us flatteringly if we continue to delay the optimal and inevitable needlessly, now that it is clearly within our reach". Besides making the whole scientific Italian legacy available for all, this tendency would permit a "multidimensional evaluation" of the research activity, not limited, as it currently happens, to considering impact factor journals, but extended to all research products from monographs to patents to research projects.

Some studies show evidence that open access journal articles are cited more and quicker and are downloaded more often [1,33]. Besides the advantage of an increasing citation rate, other criteria to be considered for an objective evaluation of research papers are the number of the article downloads and the received comments to an article. The scientific production in terms of published items could be linked to the authors' institutions and to their curricula. This would consent to give major visibility to specialties and professional qualities of the individual scientists, thus spreading awareness on the human and financial resources to be invested in the innovative branches of research and in new collaborations, avoiding the duplication of efforts and the reiteration of research studies.

## Conclusion

The digital archive set up by the ISS, DSpace ISS, represents a real opportunity to make Italian research output in the field of public health freely accessible online, beyond the traditional "colonial" dependence from foreign indexing services and databases. DSpace ISS relies on a steady structure of metadata including also Medical Subject Headings (MeSH) adopted by PubMed for subject indexing.

It is desirable that the gap due to poor information infrastructure development within the *Italian National Health Service *be finally filled in Italy. Strategic tools as institutional repositories are able to ensure appropriateness in health care delivery and to favour a decisive development of research through the access and exchange of knowledge. Another aspect should be considered: electronic items are much more exposed to "weather conditions" of our virtual time than the paper based ones. A publishing house which ceases its activity may entail the loss of its electronic archive, thus the loss of all the scientific heritage stored in it. Hence, the importance of the archiving procedures in institutional repositories in order to safeguard the knowledge. Due to their non-commercial nature, these online deposits tend to be more stable and their contents are available for free reproduction on a print basis for long lasting. Peter Suber, one of the founder of the open access paradigm, states: " *So far, paper is the only commonly used medium that we know can preserve text for hundred of years" *[34].

## Competing interests

The authors declare that they have no competing interests.

## Authors' contributions

EP, GC, IT and CDB designed the questionnaire (see Appendix), processed and described the data resulting from the survey. All authors participated in the work for appropriate portions of the content and approved the final version of the manuscript.

## Appendix

### Questionnaire Institutional repositories of the Italian Scientific Institutes for Research, Hospitalization and Health Care (IRCCS) in the field of oncology

Pilot survey edited by the Questionnaire Working Group:

G. Cognetti, E. Poltronieri, C. Di Benedetto, I. Truccolo

### Survey Promotion

This questionnaire aims to gather information on collecting information methodologies, preservation techniques, assessment and access strategies to scientific literature produced by IRCCS institutions in the field of oncology.

### Target audience

Chief librarians or professionals acting in other units of the institution in charge of managing scientific publications in the IRCCS.

### Objectives

The survey aims to:

- explore the organization, collection methods, preservation techniques and contents of the archiving systems in use to describe scientific literature;

- launch a feasible plan concerning the adoption of standard procedures for the aggregation of free-access scientific resources in the field of biomedicine, through the digital platform provided by DSpace ISS http://dspace.iss.it/dspace/.

### Survey results

The results of the questionnaire, processed by the Questionnaire Working Group solely for statistical purposes, will be reported in a paper hosted by an open access journal.

### Working Group contacts

Gaetana Cognetti (Istituto Regina Elena, Roma. Biblioteca digitale "R. Maceratini" e Biblioteca del Paziente cognetti.bib@ifo.it)

Elisabetta Poltronieri (Istituto Superiore di Sanità, Roma. Settore Attività Editoriali elisabetta.poltronieri@iss.it)

Corrado Di Benedetto (Istituto Superiore di Sanità, Roma. Settore Informatico corrado.dibenedetto@iss.it)

Ivana Truccolo (Centro di Riferimento Oncologico, itruccolo@cro.it)

### Questionnaire

1. **Name of the Institution:**_____________________________________________

2. **Which Unit of your institution is in charge of the digital repository?**

Library [ ] Other Unit [ ] Specify________________________

3. **Your institutional repository includes:**

• Journal articles with an impact factor or without an impact factor [ ]

• Journal articles with an impact factor [ ]

• Institutional series (journals, technical reports, newsletters) [ ]

• Books or book chapters [ ]

• Conference proceedings (abstracts or papers) printed or online [ ]

• Posters (image format) [ ]

• Training material (texts or abstracts of the lessons delivered, Conference programs, slides, PPT presentations) [ ]

• Clinical trials [ ]

• Guidelines [ ]

• Educational material for patients [ ]

• Patents [ ]

• Research projects approved and funded (summaries or reports) [ ]

• Other material [ ] Specify_______________________________

*4. ***The material deposited in the repository is archived as ***(mark both options, if so)*

Bibliographic citation [ ] Full text [ ]

4.1 The archive format used is: print [ ] digital [ ]

4.2 If it is only print, describe how data are structured:

4.3 If it is digital, specify the software in use:

Word [ ] Excel [ ] Access [ ]

Software tools for publishing and managing bibliographies (RefWorks, Reference Manager, etc.) [ ] Specify________________________________________________

In-house software [ ]

Other software [ ] Specify__________________________________________

5. **Type of metadata (bibliographic data) used in the repository:**

• Title [ ]

• First author or editor [ ] *Specify the format of data stored (Mario Rossi, M. Rossi, M Rossi, Rossi Mario, Rossi M)*

___________________________________________________________________

• Two separated fields, respectively for Name and Surname, are provided: yes [ ] no [ ]

• All the authors or editors (corporate authors - as working or study groups - included) [ ]

• Other kinds of authors' listings (i.e. only the first 6 authors followed by *et al*) [ ]

• Title of the publication (journal, book, conference proceedings, technical report) [ ]

• Year [ ]

• Volume [ ]

• Issue [ ]

• Pages [ ]

• Abstract [ ]

• ISSN [ ]

• ISBN [ ]

• DOI [ ]

• PMID (PubMed identifier) [ ]

• URL [ ]

• Conference name/title [ ]

• Conference venue [ ]

• Conference date [ ]

• Publisher [ ]

• Publication language [ ]

• Publication type (journal article, book, book chapter, conference paper, etc.) [ ]

• Journal impact factor

Specify : raw impact factor [ ] rank-normalized impact factor [ ]

• Indexing terms as text words [ ]

• Indexing term as descriptors of a controlled vocabulary (classification, subject headings, thesaurus - i.e. MeSH term) [ ]

Specify the terminology in use___________________________________

• Research line to whom the publication is related [ ]

6. **Besides data of the institutional repository transferred to the Italian Ministry of Health workflow, they are accessible:**

• The Scientific Directorate of the Institution only [ ]

• The internal research staff of the Institution only [ ]

• Freely on Internet [ ]

7. **Does your Institution publish any institutional series (i.e. official scientific journal, newsletter,..) ?**

yes [ ] no [ ] - If yes:

Title___________________________________________________________________

Title___________________________________________________________________

Title___________________________________________________________________

7.1 Specify whether the publications are open access (free access on Internet)

yes [ ] no [ ] - If yes:

Enter the website address________________________________________________

8. **Does your Institution agree to make its own scientific production freely accessible online on the open archive DSpace ISS **http://dspace.iss.it/dspace** set up by the Istituto Superiore di Sanità? **yes [ ] no [ ]

*9. *Please leave here any comments or notes if needed to clarify the answers given (by specifying the number of the related answer):

Name and signature of the chief librarian or the person in charge at managing the publications produced by your Institution

Name_______________________________________________________________________

Signature____________________________________________________________________

Tel._________________________________________________________________________

E-mail_______________________________________________________________________

Date ________________________________________________________________________

*Print the questionnaire and send it to*_____________________fax number _______________

within_____________________________

Thank you

### PRIVACY POLICY

Notice provided according to the terms of art. 13 of Italian Legislative Decree no. 196 of 30 June 2003 for the protection of personal data

The data provided in the Questionnaire will be processed by means of automated equipment, only to fulfill the following tasks: to build up a unique reference access point to scientific information produced by the institutions surveyed through the digital archive DSpace ISS http://dspace.iss.it/dspace/.

Does the user grant her/his permission to processing their personal data according to the above mentioned tasks?

Yes [ ] No []
